# Could miR-34a Inhibition be Used as a Tool to Overcome Drug Resistance in MCF-7 Cells Treated with Synthesized Steroidal Heterocycles?

**DOI:** 10.31557/APJCP.2021.22.3.819

**Published:** 2021-03

**Authors:** Shaymaa M M Yahya, Mervat M Abd-Elhalim, Abdou O Abdelhamid, Emad F Eskander, Ghada H Elsayed

**Affiliations:** 1 *Department of Hormones, Medical Research Division, National Research Centre, Dokki, Cairo, Egypt. *; 2 *Department of Chemistry, Faculty of Science, Cairo University, Cairo, Egypt. *

**Keywords:** Breast cancer, progesterone derivatives, miR-34a inhibitor, drug resistance, apoptotic and angiogenic genes

## Abstract

**Background:**

Progesterone derivatives have explored an improved effect on human cancer cells through combination of the explored heterocycles with progesterone moiety.miRNAs have an important role in moderating cancer cell survival, proliferation and drug resistance. The current study tested the hypothesis “whether miR-34a inhibitor has a negative impact on apoptosis and angiogenesis in MCF-7 cells treated with newly synthesized progesterone derivatives”.

**Methods:**

MCF-7 cells were treated with progesterone derivatives individually and in combination with miR-34a inhibitor. miR-34a expression levels were measured in MCF-7 cells treated with progesterone derivatives using QRT-PCR. MCF-7 cells treated with progesterone derivatives individually showed increased miR-34a expression levels. miR-34a deficient cells were treated with the newly synthesized progesterone derivatives, after that, apoptotic and angiogenic gene expression levels were determined using QRT-PCR. The studied genes were as follows: apoptotic (*Bcl-2, survivin, CCND1, CDC2, P53* and *P21*) and angiogenic (VEGF, Hif-1α, MMP-2, Ang-1, Ang-2, and FGF-1).

**Results:**

The results showed that miR-34a deficient MCF-7 cells treated with the newly progesterone derivatives still have promising effects on apoptotic and angiogenic genes. Besides, results revealed that miRNA-34a deficient MCF-7 cells exhibited improved effect of tested compounds in some apoptotic and angiogenic genes such as *CDC-2, MMP-2*.

**Conclusion:**

These results revealed that miR-34a inhibitor did not have remarkable negative effect on apoptosis and angiogenesis. On contrary, it showed an improved effect on some genes. And consequently, miR-34a inhibitor could be used safely as a tool to tackle drug resistance in breast cancer cells.

## Introduction

Breast cancer was found to account for 22% of newly discovered cancer cases each year in women. Besides, it is the most leading cause of mortality and the second most commonly diagnosed cancer (DeSantis et al., 2014). Drug resistance represents the major cause for unsuccessful chemotherapeutic strategies (Pasquier et al., 2011). Etoposide, doxorubicin, paclitaxel, topotecan, and 5-fluorouracil are commonly used chemotherapeutic agents for breast cancer; however, multidrug resistance can develop against these agents. It was reported that MicroRNAs (*miRNAs*) are key players in drug resistance (Yu et al., 2015). *miRNAs* have pivotal roles in moderating cancer cell survival, proliferation and drug resistance development, however, the exact mechanisms of chemotherapy response control by *miRNAs *needs further investigations and their therapeutic benefits have not been fully evaluated (Yahya et al., 2014).

Recently, microRNA-34a (miR-34a) has attracted extensive research interests due to its involvement in myriad of oncogenic pathways in different cancers (Ito et al., 2017; Wen et al., 2017; Shi et al., 2014; Adams et al., 2016; Li et al., 2014a; Li et al., 2014b). Besides, it is a candidate for diagnostic as well as a prognostic biomarker (Imani et al., 2017; Raptiet al., 2017; Chen et al., 2017). In a recent work (Yahya et al., 2017), our group introduced newly synthesized pyridine, thiazole, thiazolopyridine, pyrazole, and pyrazolopyridine progesterone derivatives. These compounds were prepared and tested for their anti-proliferative effects where they proved a noticeable anti-proliferative action on breast cancer cells. This study found that compounds 2, 3, 4, 6, 7, 8 and 9 affected positively on apoptosis by the suppression of *Bcl-2*, however, survivin and *CCND1 *expression levels were down regulated by compounds 3, 4, 6, 7, 8, 9. Moreover, Compound 4 enhanced the apoptosis process by the enhancement of *P53* gene expression levels. Concerning the angiogenic process, these compounds affected angiogenesis by the suppression of *VEGF, Ang-2, MMP-9 *and* FGF-1*; and the enhancement of HIF-1α and Ang1. 

Interestingly, in a previous work of our research group (Yahya et al., 2018), it was reported that *miR-34a *expression levels were up-regulated upon treating MCF-7 cells with modified steroid derivatives as a simultaneous response to drug resistance. Besides, it was previously reported that overexpression of *miR-34a* inhibited the proliferation, migration, and invasion of breast cancer cells (Avtanski et al., 2016). It also modulates drug sensitivity of breast cancer by affecting some of anti-apoptotic genes such as *BCL-2 *and *CCND1*. However, Kastl et al., (2012) reported that *miR-34a* over expression is associated with docetaxel resistance and enhance breast cancer stemness and drug resistance (Kim et al., 2016), on contrary, by having NOTCH 1 and PRKD1 as its targets, miR-34a affect chemoresistance of breast cancer cells to adriamycin (Li et al., 2012). The former findings together motivated us to test the hypothesis whether knocking down miR-34a will have a negative impact on apoptotic and angiogenic pathways in cells treated with the newly synthesized progesterone derivatives. 

## Materials and Methods

*Cell propagation, maintenance and treatment*

**Figure 1:**
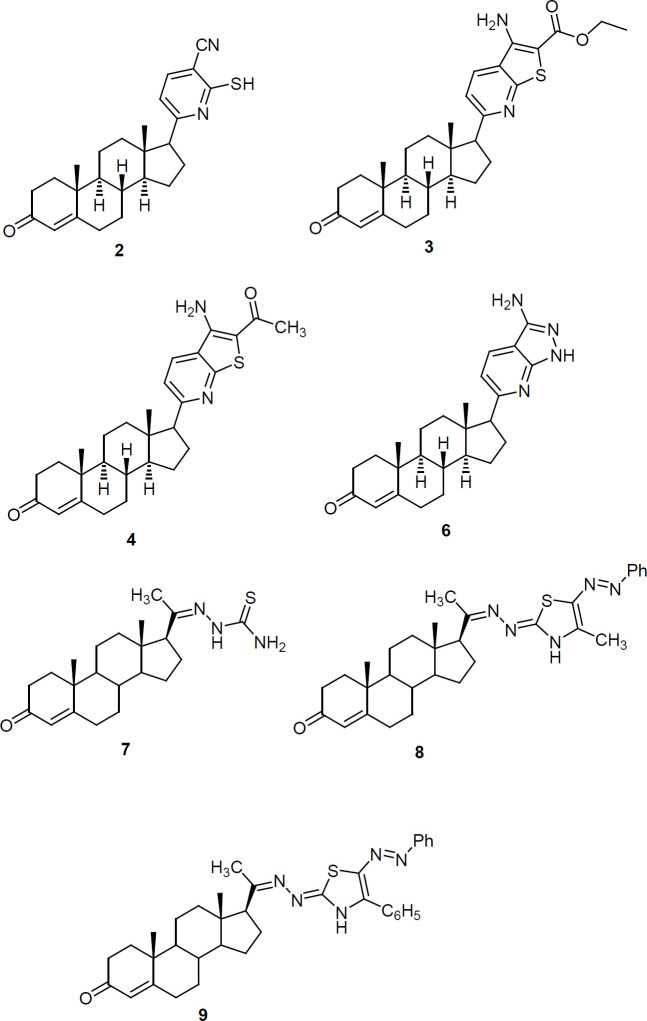
Chemical Structure of the Newly Synthesized Heterosteroids (Yahya et al. 2017).

Breast cancer MCF-7 cells were purchased from ATCC (American Type Culture Collection) and maintained in the proper conditions. The cells were cultured in Dulbecco’s modified Eagle’s Medium (DMEM) (Lonza, Beligium) supplemented by 10 % fetal bovine serum (FBS), 4 mM L-glutamine, 100 U/ml penicillin, and 100 μg/ml streptomycin sulfate at 37˚C in a humidified incubator with 5 % CO_2_. The cells harvested after trypsinization (0.025 % trypsin and 0.02 % EDTA) and washed twice with Dulbecco’s phosphate-buffered saline (DPBS) (Yahya et al., 2016; Hamed et al., 2018). When the cells density reached approximately 80%, cells were split for further culture. The experiments were made up when the cells were in the logarithmic growth phase. Before transfection, 1.5x10^5^ cells were seeded per well of two 24-well plate in 1.5 ml of DMEM culture medium containing 10% FBS serum and antibiotics. The second day, cells were transfected with 100nM miR-34a-5p inhibitor (Qiagen, USA) in a serum free media using 1µl of the Hiperfect transfection reagent. MiScript Inhibitor negative control which has no homology to any known mammalian gene was used as negative control. The third day, the media were changed with fresh complete media and the cells in the 1^st^ plate were incubated at the proper conditions for another 48 hrs with the tested compounds (Yahya et al., 2016; Hamed et al., 2018). While, the cells in the 2^nd^ plate were subjected to *miRNA* extraction to verify successful transfection. These compounds were synthesized and subject to cytotoxicity analysis in a previous work (Yahya et al., 2017). The cells were incubated with various concentrations of the test compounds (6.25, 12.5, 25 and 50 μM) for 48 h at a cell density of 104 cells/well of 96 well plate. Using the relation between used concentrations and neutral red intensity value, IC_50_ of tested compounds was calculated. Four parameter equation logistic curve was used (log concentration vs. % cell growth as compared to control cells). The structures of these compounds are illustrated in Figure 1 ( Yahya et al., 2017) and their IC_50_ values are listed in Table 1.

*Quantitative miRNA determination*

*miR-34a *expression levels were determined using the Qiagen miscript system (Qiagen, USA) according to manufacturer instructions. miRNeasy kit was used for Purification of RNA containing *miRNA*, after that cDNA generation from RNA containing *miRNA* was performed using miscript RT-kit. Real-Time PCR was used for detection of mature *miRNA* using *miRNA*-specific primers to hsa-miR-34a. The cycling conditions were as follow: denaturation for 15 s at 94ºC, annealing for 30 s at 55ºC and extension for 30 s at 70ºC. Fluorescence data were collected using MiniOpticon Bio-Rad Real Time Thermal Cycler.

*RNA isolation, clean up and Quantitative real time RT-PCR*

RNA was isolated using Qiazol buffer (Qiagen, USA) according to manufacturer instructions. RNA was subsequently cleaned up using RNAeasymini Kit (Qiagen, USA). *β-actin, BCL-2, survivin, CCND1, CDC2, P53* and *P21, VEGF, HIf-1α, MMP-2, Ang-1, Ang-2,* and *FGF-1* genes copy numbers were quantified using QuantiFastSybergreen RT-PCR. The copy numbers were normalized to 100,000 copies of the house keeping *beta-actin* gene. Primer sequences are listed in Table 2. The RT and subsequent PCR cycling conditions were as follow, 50ºC for 10 min, 95ºC for 5 min, 95ºC for 15s, then 60ºC for 30 s, the number of cycles were 40 cycles. MiniOpticonTM Bio-Rad Real Time Thermal Cycler was used for gene expression quantitation.

*Statistical analysis*

The data were analyzed using Microsoft Excel. All the data are expressed as Mean ± standard error mean. Analysis of the data was done using student t-test to detect the significant difference between the studied compounds. A level of P < 0.05 was defined as statistically significant. All data were reproducible.

## Results

**Figure 2:**
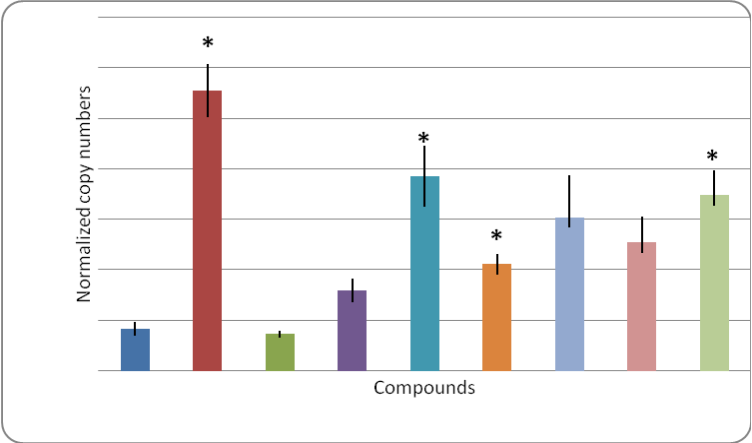
Effect of Tested Compounds on miRNA-34a Expression Levels. C, Control cells; Tx, Tamoxifen treated cells; Data are represented as mean ±SE, Data were reproducible, *P<0.05

**Figure 3:**
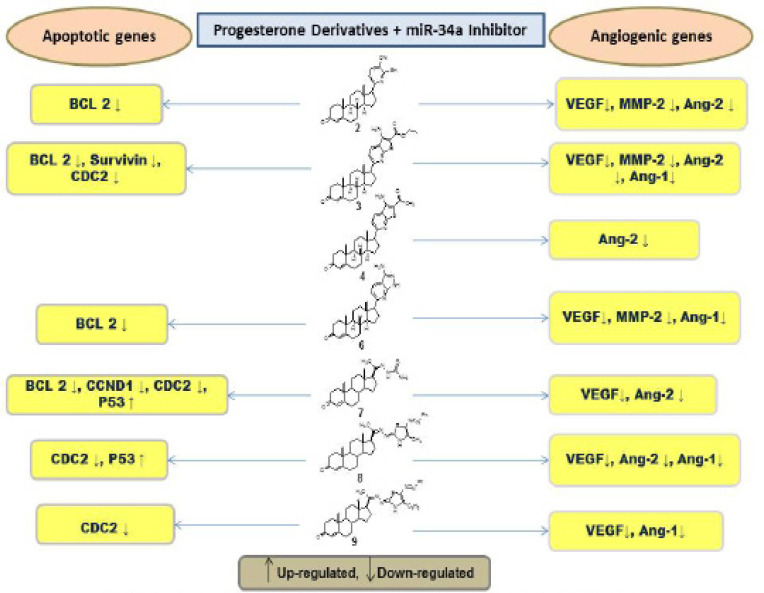
The Effect of miR-34a Inhibition on Progesterone Derivatives Treated MCF-7 Cells

In the current work, all tested progesterone derivatives significantly elevated the expression levels of *miR-34a *(Figure 2). *MiRNA*-34a expression level was reduced to 7.42% as compared to negative control treatment. The effect of miR-34a inhibition on progesterone derivatives treated MCF-7 cells is summarized in Figure 3. in a previous work of our research group all tested progesterone derivatives (compounds 2, 3, 4, 6, 7, 8, and 9) revealed noticeable down-regulation of *BCL-2* gene expression level as compared to control cells (Yahya et al., 2017). However, MCF-7 cells treated with tamoxifen showed only a non-significant reduction on *BCL-2* expression level. On the other hand, treating MCF-7 cells with miR-34a inhibitor synergistically with tamoxifen and progesterone derivatives, reduced *BCL-2* expression level in case of tamoxifen, compounds 2, 3, 6, and 7. However, this reduction was not significant in case of compound 8 and 9 treatments (Table 3). Yahya et al., (2017) showed that treating MCF-7 cells with compounds 3, 4, 6, 7 and 9 dramatically reduced survivin expression level. However, upon knocking down miR-34a only compounds 2 and 3 significantly reduced *survivin* expression levels. Besides, tamoxifen, compounds 6, 7, and 9 resulted in a non-significant reduction in survivin levels (Table 3). Similarily, treating MCF-7 with miR-34a inhibitor synergistically with compounds 7 and tamoxifen resulted in reduction in *CCND1* expression level while compounds 2, 4, 6, 7, 9 produced a non significant reduction in this gene expression levels (Table 3). Previously (Yahya et al., 2017), treating MCF-7 cells with compounds 3, 4, 8 individually resulted in significant reduction in *CCND1* gene expression levels, meanwhile compounds 6, 7, and 9 resulted in non-significant reductions in the expression levels of* CCND1* gene. Interestingly,* CDC-2* gene expression was dramatically decreased in* miR-34a *suppressed *MCF-7 *cells treated with compounds 3, 7, 8, 9, in addition to a non significant reduction in cells treated with tamoxifen, compounds 2, 4, and 6 (Table 3). *P53* as an important tumor suppressor gene was only up regulated in MCF-7 cells treated with compound 4 individually; however, upon synergistically inhibiting miR-34a, P53 was significantly up regulated in MCF-7 cells treated with tamoxifen, compounds 7, and 8 (Table 4). The current findings suggest that miR-34a inhibition did not affect the promising action of the novel compounds in rising *P53* expression levels. *P21* gene expression level was not affected in MCF-7 cells subjected to novel compounds individually. However, it was down regulated in MCF-7 treated with tamoxifen and miR-34a inhibitor (Table 4). In the current study, MCF-7 treated with compounds 2, 3, 6, 7, 8, 9, alongside with miR-34a inhibitor significantly resulted in down regulation in *VEGF* expression levels, and only compound 4 and tamoxifen did not show this effect (Table 4). MCF-7 cells treated with *tamoxifen*, compounds 2 and 9 showed significant elevation in *HIF-1 alpha* expression levels (Yahya et al., 2017), in contrary, when miR-34a was inhibited, cells treated with compounds 3, 6, 7, 8, 9 showed significant down regulation in this gene expression levels (Table 4). In the current study, MCF-7 cells treated with the tested compounds alone did not produce significant reduction in *MMP-2* expression levels, however, when it was treated with miR34a inhibitor alongside with tamoxifen, compounds 2, 3, 6, 9, *MMP-2* expression levels was dramatically reduced (Table 5). This effect could be attributed to the observed reduction in HIF-1alpha in miR-34a knocked cells. 

MCF-7 cells treated with miR-34a inhibitor and compounds 3, 6, 8 and 9 resulted in significant reduction in *Ang-1* expression levels which is consistent with its physiological role as a promoter of angiogenesis, however its levels were significantly over expressed when treated with tamoxifen, compounds 2, 7, and 9 individually (Table 5) (Yahya et al., 2017). Similarly, all tested compounds showed significant reduction in *Ang-2* expression levels except compounds 6 and 9 (showed a non-significant reduction), which is relatively comparable to Ang-2 levels when treating cells with the tested compounds individually (all tested compounds showed significant reduction in *Ang-2* expression levels (Table 5). 

FGF-1 which was found to mediate tumor initiation, progression and metastasis, did not show significant changes in its expression levels when treating *MCF-7* cells with miR-34a inhibitor with the tested compounds (Table 5).This opposes what happened when this cells was treated with compounds 2, 3, and 8 individually, as *FGF-1* expression levels were significantly reduced (Yahya et al., 2017). This could suggest possible role of miR-34a in modulating FGF-1 levels which needs further investigations.

**Table 1:** The in vitro Cytotoxic Activity of the Newly Synthesized Compounds on MCF-7 Cancer Cell Line

Compd.No	IC_50_ (µM)
4.12	2
4.71	3
5.87	4
5.28	6
3.82	7
5.28	8
5.87	9
3.53	Tamoxifen

IC_50_, Concentration required to inhibit cell viability by 50%

**Table 2:** Primers Used for Apoptotic and Angiogenic Pathway Analyses

Gene	Primer forward (5'-3')	Primer reverse (5'-3')
*β-actin*	CCTTCCTGGGCATGGAGTCCT	GGAGCAATGATCTTGATCTTC
*BCL-2*	CCTGGTGGACAACATCGCC	AATCAAACAGAGGCCG CATGC
*Survivin*	AGGACGGCCCTTCTTGGAGG	CTTTTTATGTTCCTCTATGGGGTC
*CDC2*	CAAATATAGTCAGTCTTCAGGATG	CCTGTAGGATTTGGTATAAATAAC
*CCND1*	GAGGAAGAGGAGGAGGAGGA	GAGATGGAAGGGGGAAAGAG
*P53*	AGA GTC TAT AGG CCC ACC CC	GCT CGA CGC TAG GAT CTG AC
*P21*	AAG ACC ATG TGG ACC TGT	GGT AGA AAT CTG TCA TGC TG
*VEGF*	TACCTCCACCATGCCAAGTG	ATGATTCTGCCCTCCTCCTTC
*HIF-1α*	GCAAGCCCTGAAAGCG	GGCTGTCCGACTTTGA
*MMP-2*	ATGCTTCCAAACTTCACGCTCT-	CAGGGTTTCCCATCAGCATT
*Ang-1*	AAAGGTCACACTGGGACAGC	TTCTGACATTGCGCTTTCAA
*Ang-2*	TCCAAGCAAAATTCCATCATTG	GCCTCCTCCAGCTTCCATGT
*FGF-1*	AAGCCCGTCGGTGTCCATGG	GATGGCACAGTGGATGGGAC

**Table 3:** Effect of miRNA-34a Inhibition Synergistically with Tested Compounds on *BCL-2, survivin, CCND1,* and *CDC2* Gene Expression Levels. NC, MiScript Inhibitor negative control treated cells; Tx, Tamoxifen treated cells. Data are represented as mean ±SE; Data were reproducible, *P<0.05

Genes	Normalized copy numbers of *BCL-2 *gene	Normalized copy numbers of *survivin *gene	Normalized copy numbers of *CCND1* gene	Normalized copy numbers of *CDC2* gene
Compounds				
*NC*	16323.3±1328.9	12566.7±1315.19	4362574.5±531367.73	764621.9±111428.39
*Tamoxifen*	3670.5±956.1*	12146.7±653.72	92745.0±7873.84*	455488.6±45366.17
2	6163.4±624.1*	4346.4±573.41*	3528464.9±605266.78	657286.3±69796.17
3	4883.3±69.3*	310.3±18.69*	4405250.9±704416.75	380827.8±65095.22*
4	41337.6±5169.3*	15200.7±5940.53	3872463.5±457621.15	560245.7±75715.85
6	5108.9±134.5*	12518.6±1397.87	2762058.4±247331.67	369646.7±22845.35
7	5170.9±498.5*	11463.4±870.863	2140033.8±45209.63*	186787.3±8259.18*
8	11992.7±1415.9	13340.2±387.49	3684999.0±2205126.08	105688.1±5640.14*
9	11562.1±651.9	10830.6±365.13	4135436.5±1894751.06	241498.9±35490.38*

**Table 4:** Effect of miRNA-34a Inhibition Synergistically with Tested Compounds on *P53, P21, VEGF,* and *Hif-1α *Gene Expression Levels. NC, MiScript Inhibitor negative control treated cells; Tx, Tamoxifen treated cells; Data are represented as mean ±SE, Data were reproducible, *P<0.05

Genes	Normalized copy numbers of *P53* gene	Normalized copy numbers of *P21* gene	Normalized copy numbers of *VEGF* gene	Normalized copy numbers of *Hif-1α* gene
Compounds				
*NC*	87.8±9.0	676652.4±46463.56	10329335.2±515018.4	271226.8±42606.55
*Tamoxifen*	894.3±129.5*	926948.7±67943.13*	10926537.0±473735.0	13281.9±474.05*
2	63.5±8.3	385834.4±47514.07*	5833701.1±875074.4*	173003.7±22458.14
3	25.1±1.6*	117823.5±15648.62*	3388792.0±449572.7*	47305.7±917.46*
4	65.3±6.1	331034.8±9472.42*	31763403.8±2217195.4*	283276.9±24351.39
6	58.4±12.3	248011.9±29900.24*	4377939.5±446935.4*	84645.5±6637.07*
7	183.3±1.7*	160652.9±4757.99*	1821693.0±299328.1*	17937.7±1274.72*
8	259.0±25.3*	185973.0±42125.24*	6861655.5±663298.0*	110716.7±10815.01*
9	241.8±50.8	274048.4±13694.67*	7543390.1±984681.7	42780.5±3173.73*

**Table 5:** Effect of miRNA-34a Inhibition Synergistically with Tested Compounds on *MMP-2, Ang-1, Ang-2*, and *FGF-1* Gene Expression Levels. NC, MiScript Inhibitor negative control treated cells; Tx, Tamoxifen treated cells; Data are represented as mean ±SE, Data were reproducible, *P<0.05

Genes	Normalized copy numbers of *P MMP-2* gene	Normalized copy numbers of *Ang-1* gene	Normalized copy numbers of *Ang-2* gene	Normalized copy numbers of *FGF-1* gene
Compounds				
*NC*	72.6±4.44	496.4±22.55	4956.5±276.73	4167.8±767.01
*Tamoxifen*	11.4±1.83*	1009.9±275.98	4574.1±153.74	3592.6±329.86
2	12.2±1.59*	399.9±62.41	3023.7±359.85*	3004.3±277.34
3	3.7±0.21*	95.2±4.71*	1306.0±114.46*	1511.7±61.89
4	67.5±20.19	454.4±34.13	2206.1±137.71*	4096.2±465.23
6	1.0±0.17*	224.5±19.25*	4182.5±150.18	1966.3±111.67
7	64.6±5.21	465.9±57.72	1421.2±84.55*	4613.5±52.51
8	79.7±9.26	160.3±22.45*	1237.9±159.17*	6583.1±1264.45
9	110.7±5.41*	167.4±28.23*	3728.3±990.12	2843.8±188.01

## Discussion

Many *miRNA*s were found to be a key player in the progression of breast cancer chemoresistance by regulation of apoptosis, estrogen resistance, drug transporters modulation, epithelial mesenchymal transition (EMT), and cancer stem cells (Yalcin and Gunduz, 2016). Apoptosis is a very important mechanism which is responsible for eliminating abnormal cells in normal tissues as well as cancerous tissues (Kaplan et al., 2020). The positive role of miR-34a in antagonizing malignancy consequences, like the down regulation of cancer cell differentiation, proliferation, invasion and migration is well established (Saito et al., 2015; Imani et al., 2017; Wang et al., 2017). The definite role played by miR-34a in breast cancer has many controversies. Indeed miR-34a may affect positively in the modulation of drug sensitivity in breast cancer by suppression of NOTCH1, BCL-2, and CCND1 (Li et al. 2017). Kastl et al., (2012) reported that *miR-34a *over expression is associated negatively with docetaxel resistance and drug sensitivity could be restored upon miR-34a modulation. 

In the current work, all tested progesterone derivatives significantly up--regulated the expression levels of *miR-34a* which is consistent with our previous findings (Yahya et al., 2018) where the treatment of MCF-7 cells with steroid derivatives significantly up regulated *miR-34a *expression levels. This was attributed to the evoked drug resistance against tested compounds 

It is well established that miR-34a targets BCL-2 and various cyclins (Li et al., 2013).However knocking down miR-34a did not affect on BCL-2 reduction by the tested compounds. This means that the knocking down of miR-34a did not affect negatively on the promising effect of the most of novel derivatives on *BCL-2* gene expression levels. Yahya et al., (2017) showed that treating MCF-7 cells with compounds 3, 4, 6, 7 and 9 dramatically reduced survivin expression level. However, upon knocking down miR-34a only compounds 2 and 3 significantly reduced *survivin *expression levels. Besides, tamoxifen, compounds 6, 7, and 9 resulted in a non-significant reduction in survivin levels. miR-34a was found to target survivin. This effect could be indirect regulation through the downregulation of survivin upstream activators or transcriptional factors, which are targets of miR-34a; or Direct regulation (Huang et al., 2015). Moreover, over expression of* miR-34a *significantly down regulated survivin in HNSCC cell line, UM-SCC-74A (Kumar et al., 2012), in non-small cell lung cancer (NSCLC) cells (Ji et al., 2012), laryngeal squamous cell carcinoma cell lines (Cao et al., 2013), a gastric cancer cell line (Chen et al., 2010), murine melanoma cells (Shen et al. 2012). Interestingly, *CDC-2* gene expression was significantly down regulated in MCF-7 cells treated with miR-34a inhibitor synergistically with compounds 3, 7, 8, 9, in addition to a non significant reduction in cells treated with tamoxifen, compounds 2, 4, and 6 This reduction was not achieved in MCF-7 cells treated with these compounds individually. Chen et al., (2013) found that CDK1 (encoded by *CDC2* gene) inhibition-induced cell death in neuroblastoma cells was dependent on the suppressed MYCN levels and miR-34a-mediated. Where the down regulation of MYCN resulted from CDK1 inhibition which consequently decreased the transcriptional activation of MYCN on the survivin promoter. CDK1 inhibition resulted in elevation of miR-34a, meanwhile *miR-34a* suppression up-regulated the expression of *MYCN* and enhanced cell survival of neuroblastoma cells treated with CDK1 antagonist. These findings may interpret the promising effect achieved in CDC2 reduction upon miR-34a inhibition. *P53* as an important tumor suppressor gene was only up regulated in MCF-7 cells treated with compound 4 individually; however, upon synergistically inhibiting miR-34a, P53 was significantly up regulated in MCF7cells treated with tamoxifen, compounds 7, and 8. The *miR-34a* gene is located at lp36.23. MiR-34a was firstly introduced as a target of P53 which acted as an important tumor suppressor (He et al., 2007; Misso et al., 2014). Ye et al., (2016) stated that the anti-tumor effect of miR-34a was originally dependent on the modulation of SIRT1 and p53/p21 protein and not apoptosis-related proteins. In our previous study (Yahya et al., 2017); it was found that all tested progesterone derivatives produced significant down regulation in vascular endothelial growth factor (*VEGF*) expression levels. *VEGF* was identified as a target gene for* miR-34a* (Yu et al., 2014). In the current study, MCF-7 treated with compounds 2, 3, 6, 7, 8, 9, alongside with miR-34a inhibitor significantly resulted in down regulation in *VEGF* expression levels, and only compound 4 and tamoxifen did not show this effect. MCF-7 cells treated with tamoxifen, compounds 2 and 9 showed significant elevation in *HIF-1 alpha* expression levels, in contrary, when miR-34a was inhibited, cells treated with compounds 3, 6, 7, 8, 9 showed significant down regulation in this gene expression levels. This could be explained by the findings of Lin et al., (2017) who found that miR-34a up regulation significantly up regulates HIF-1alpha and down regulates SIRT1 and consequently promoted apoptosis. Previous observations found that hypoxia and HIF-1 enhances the expression levels and/or activities of *MMP-2* and *MMP-9* (Krishnamachary et al., 2006; Munoz-Najar et al., 2006). In the current study, MCF-7 cells treated with the tested compounds alone did not produce significant reduction in *MMP-2* expression levels, however, when it was treated with miR34a inhibitor alongside with tamoxifen, compounds 2, 3, 6, 9, *MMP-2 *expression levels was dramatically reduced. This effect could be attributed to the observed reduction in HIF-1alpha in miR-34a knocked cells. FGF-1 which was found to mediate tumor initiation, progression and metastasis; did not show significant changes in its expression levels when treating *MCF-7* cells with miR-34a inhibitor with the tested compounds. This opposes what happened when this cells was treated with compounds 2, 3, and 8 individually, as *FGF-1* expression levels were significantly reduced. This could suggest possible role of miR-34a in modulating FGF-1 levels which needs further investigations.

MCF-7 cells treated with miR-34a inhibitor and compounds 3, 6, 8 and 9 produced significant reduction in *Ang-1* expression levels which is consistent with its physiological role as a promoter of angiogenesis, however its levels were significantly over expressed when treated with tamoxifen, compounds 2, 7, and 9 individually. Similarly, all tested compounds showed significant reduction in *Ang-2 *expression levels except compounds 6 and 9 (showed a non-significant reduction), which is relatively comparable to Ang-2 levels when treating cells with the tested compounds individually (all tested compounds showed significant reduction in *Ang-2 *expression levels. Unfortunately, little is known about the role of miR-34a in modulating Angiopioteins which needs further studies to be elucidated.

In conclusion, the current study revealed that *miRNA*-34a inhibitor could be used as a tool to tackle drug resistance in breast cancer. The observed results in this study points out that miR-34a inhibition did not have a noticeable negative impact on apoptosis and angiogenesis. On contrary, *miRNA*-34a deficient MCF-7 cells showed improved effect of the tested progesterone derivatives in some apoptotic and angiogenic genes such as *CDC-2*, *MMP-2*. 

*Abbreviations*

miR-34a: microRNA-34a; DMEM: Dulbecco’s modified Eagle’s Medium; FBS: fetal bovine serum; DPBS: Dulbecco’s phosphate-buffered saline; EMT: epithelial mesenchymal transition; NSCLC: non-small cell lung cancer; VEGF: vascular endothelial growth factor.

## Author Contribution Statement

YS, suggested the point of research, designed the experiment, wrote the manuscript, and participated in the statistical analysis of the data. EG, participated in the design of the experimental work, participated in writing the manuscript, and participated in the statistical analysis of the data. AM, participated in the design of the experimental work and writing the manuscript. AA, participated in the design of the experimental work and writing the manuscript. EE, revised the manuscript. The authors read and approved the final manuscript.
